# Prevalence, risk factors, and healthcare‐seeking among subjects with esophageal symptoms: A community‐based study in a rural Bangladeshi population

**DOI:** 10.1002/jgh3.12417

**Published:** 2020-09-22

**Authors:** M. Masudur Rahman, Uday C Ghoshal, Md. Golam Kibria, Nigar Sultana, Faruque Ahmed, AHM Rowshon, Mahmud Hasan

**Affiliations:** ^1^ Department of Gastroenterology Sheikh Russel National Gastroliver Institute and Hospital Dhaka Bangladesh; ^2^ Department of Gastroenterology Sanjay Gandhi Postgraduate Institute of Medical Sciences Lucknow India; ^3^ Department of Gastroenterology Delta Medical College and Hospital Dhaka Bangladesh; ^4^ Department of Gastroenterology Shaheed Suhrawardy Medical College Dhaka Bangladesh; ^5^ President, Gastroliver Foundation Dhaka Bangladesh

**Keywords:** functional esophageal disorders, gastroesophageal reflux disease, globus, heartburn, Rome criteria

## Abstract

**Background and Aim:**

As there is a scarcity of data on overall prevalence, risk factors, and health‐care utilization of esophageal symptoms using the Rome criteria in the rural population in Asia, we undertook a study with the aims to evaluate: (i) the prevalence of heartburn, chest pain, globus, and dysphagia of presumed esophageal origin; (ii) the prevalence of functional esophageal disorders by Rome III criteria; (iii) the risk factors for esophageal symptoms; and (iv) the health‐care utilization.

**Methods:**

This door‐to‐door survey was conducted in three villages (Charcharia, Churain of Dhaka district, and Kharrah of Munshiganj district of Bangladesh) among the adult population (≥18 years) using the translated and validated Enhanced Asian Rome III questionnaire.

**Results:**

Of 3559 individuals, 3351 (94.15%) responded (mean age 40.41 ± 16.04 years, female, 1924 [57.4%]). Heartburn was the most common symptom, 863 (25.8%), followed by chest pain, 367 (11%); globus, 285 (8.5%); and dysphagia, 146 (4.4%). At least one symptom was present in 1108 (33.1%) respondents. Based on Rome III criteria, 428 (12.8%), 41 (1.2%), 49 (1.5%), 26 (0.8%), and 524 (15.6%) had heartburn, chest pain, globus, dysphagia, and at least one functional esophageal disorder, respectively. Female gender, lower family income, presence of functional dyspepsia‐irritable bowel syndrome (FD‐IBS) overlap, FD only, and psychological distress were found to be risk factors for esophageal symptoms on multivariate analysis. Among the subjects with any esophageal disorders, 156 (14.1%) consulted any health‐care provider, and 517 (46.6%) took antisecretory medications.

**Conclusion:**

Esophageal symptoms are common in the rural community of Bangladesh and are associated with substantial health resource utilization.

## Graphical abstract

This door‐to‐door survey was conducted in a rural community of Bangladesh among the adult population using the translation‐validated Enhanced Asian Rome III questionnaire with the aims to study: (i) the prevalence of heartburn, chest pain, globus, and dysphagia of presumed esophageal origin; (ii) the prevalence of functional esophageal disorders by Rome III criteria; (iii) the risk factors for esophageal symptoms; and (iv) health‐care utilization. This study suggests that esophageal symptoms are common in the rural community of Bangladesh and are associated with substantial health resource utilization.

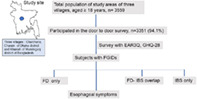

## Introduction

Esophageal symptoms are common in clinical practice.^1^ These may affect the quality of life (QoL) adversely and are associated with health‐care resource utilization and economic burden from consultation, use of medications, and absence from work.^2^, ^3^ Heartburn, chest pain, globus, and dysphagia of presumed esophageal origin may result from structural lesions, mucosal inflammation, or esophageal motor abnormalities.^4^ Endoscopy of the upper gastrointestinal tract (UGIT) with or without biopsy, pH monitoring, and esophageal manometry are often carried out to diagnose esophageal symptoms in clinical practice.^5^ Even after extensive investigations to identify structural, inflammatory, motor, or metabolic abnormalities, no cause is found in a proportion of such patients. These groups of patients are labeled as having functional esophageal disorders, which are a spectrum of functional gastrointestinal disorders (FGIDs) characterized by chronic recurrent symptoms in the absence of identifiable causes; these are currently diagnosed by Rome criteria .^6^

A handful of studies have described the prevalence of noncardiac chest pain (NCCP), gastroesophageal reflux disease (GERD), globus, or dysphagia in the general population.^4^, ^6^, ^7^, ^8^, ^9^ However, the studies that have addressed the overall frequency of esophageal disorders of presumed functional origin using Rome criteria in the community are minimal and have been reported from Western countries.^10^, ^11^ There is a scarcity of such data from Asia, particularly from the rural community where most people live. Such a study is essential as the dietary, cultural, psychological, and socioeconomic factors; health‐care systems; and religious beliefs may affect symptom burden, perceptions, QoL, health‐care utilization, and treatment.^12^, ^13^ Hence, we conducted a door‐to‐door survey among a rural community of Bangladesh with the aims (i) to study the prevalence of heartburn, chest pain, globus, and dysphagia of presumed esophageal origin; (ii) to study the prevalence of esophageal disorders of presumed functional origin based on Rome III criteria; (iii) to determine the risk factors for esophageal symptoms; and (iv) to determine the consultation rate, medications use, and QoL issues among the subjects with esophageal symptoms.

## Methods

### *Study design and population*

**Figure 1:**
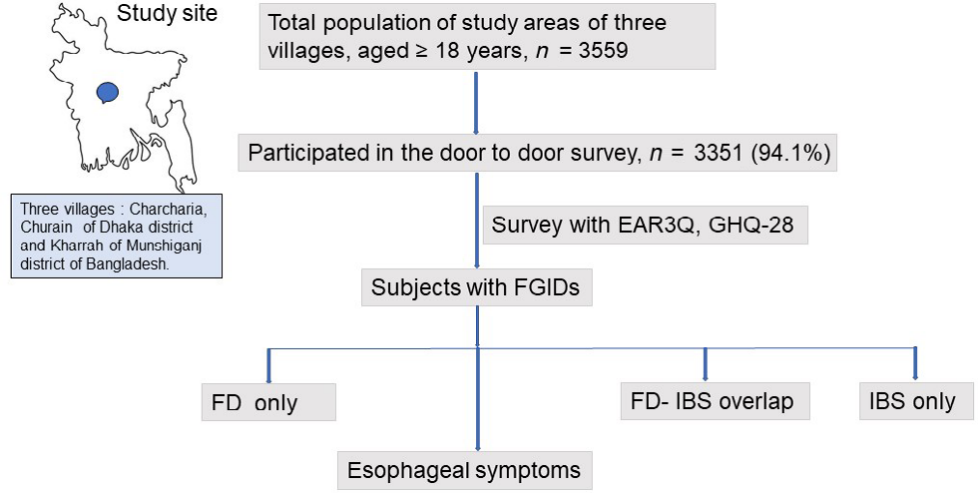
Study outline. EAR3Q, Enhanced Asian Rome 3 Questionnaire; FD, functional dyspepsia; FGIDs, functional gastrointestinal disorders; GHQ‐28, General Health Questionnaire‐28; IBS, irritable bowel syndrome.

This cross‐sectional study was performed during the period between November 2012 and November 2013 among the adult population (≥18 years) in three villages (Charcharia, Churain of Nawabganj upazila of Dhaka district and Kharrah of Srinagar upazila of Munshiganj district of Bangladesh). A manual census was conducted through a house‐to‐house survey by three trained field research assistants. Interviews of the subjects were taken using the Enhanced Asian Rome III questionnaire (EAR3Q) translated and validated in the Bengali language. During the survey, each subject filled up the questionnaire himself/herself, except when assistance was needed from the field workers due to illiteracy or visual impairment or difficulty understanding the questionnaire. Figure 1 shows the study protocol. A data entry operator entered the data, 10% of which were cross‐checked by the two investigators (M. Masudur Rahman and Nigar Sultana). The Institutional Ethics Committee approved the study protocol. Written informed consent was obtained from each study subject.

### *The questionnaire*

The questionnaire was translated and validated in Bengali. It had subsections on (i) sociodemographic information, (ii) clinical profile including EAR3Q,^14^ and (iii) General Health Questionnaire‐28 (GHQ‐28). Apart from the Rome III diagnostic questionnaire, the EAR3Q included questions related to (i) consultation with any specific health‐care providers, (ii) use of medications for the last 3 months for the concerned gastrointestinal problems, and (iii) QoL issues. QoL questions assessed to what extent a specific symptom affected the QoL of a subject. Of the five probable answers (not at all, a little, somewhat, a lot, and a great deal), the presence of “a lot” or “a great deal” was considered to demonstrate the significant impairment of QoL. GHQ‐28 was also translated and validated in Bengali. It had four subclasses: somatic symptoms, anxiety and insomnia, social dysfunction, and depression.^15^ Each question of the four domains of GHQ 28 was scored from 0 to 3. The scores of all the 28 questions were added to calculate the total score. A score of 23 or more was considered abnormal and demonstrated the presence of significant psychological distress.

### *Definitions*

The prevalence and frequency of heartburn, globus, chest pain, and dysphagia in the last 3 months were assessed using the EAR3Q. The Rome III functional esophageal disorders are functional heartburn, functional chest pain of presumed esophageal origin, functional dysphagia, and globus.^16^ Functional esophageal disorders cannot be explained by structural diseases, histopathology‐based motor disturbances, or GERD. Hence, for the diagnosis of functional esophageal disorders, endoscopy of the UGIT, pH monitoring, and esophageal manometry are required to exclude structural disorders, motor abnormalities and GERD.^6^ In the present study, only the symptom‐based conditions that fulfill the frequency threshold and the duration criteria for the diagnosis of functional esophageal disorders were applied, but no investigations, such as endoscopy, pH monitoring, or manometry, were used. Functional dyspepsia (FD) and irritable bowel syndrome (IBS) were defined according to the Rome III criteria.^17^, ^18^

### *Statistical analysis*

Normally distributed continuous data were presented as mean and standard deviation and non‐parametric data as median and interquartile range. Categorical data were presented as proportion. Normally distributed continuous data were analyzed using unpaired *t*‐test. Nonparametric continuous and categorical data were analyzed using Mann–Whitney *U* tests and Chi‐squared tests, respectively. Binary logistic regression analysis was used for the adjusted odds ratio and 95% confidence interval calculation. All the factors considered to be associated with the dependent variable on univariate analysis were entered into the logistic regression analysis. *P* values less than 0.05 were considered significant. Statistical analysis was carried out using SPSS version 15 (SPSS, Inc., Chicago, IL, USA).

## Results

The total adult population of the study areas was 3559; of them 3351 (94.15%) responded. Figure 1 shows the study enrolment. The mean age of the study subjects was 40.41 ± 16.04 years. Of 3351, 1924 (57.4%) were female.

### *Prevalence of esophageal symptoms*

**Figure 2:**
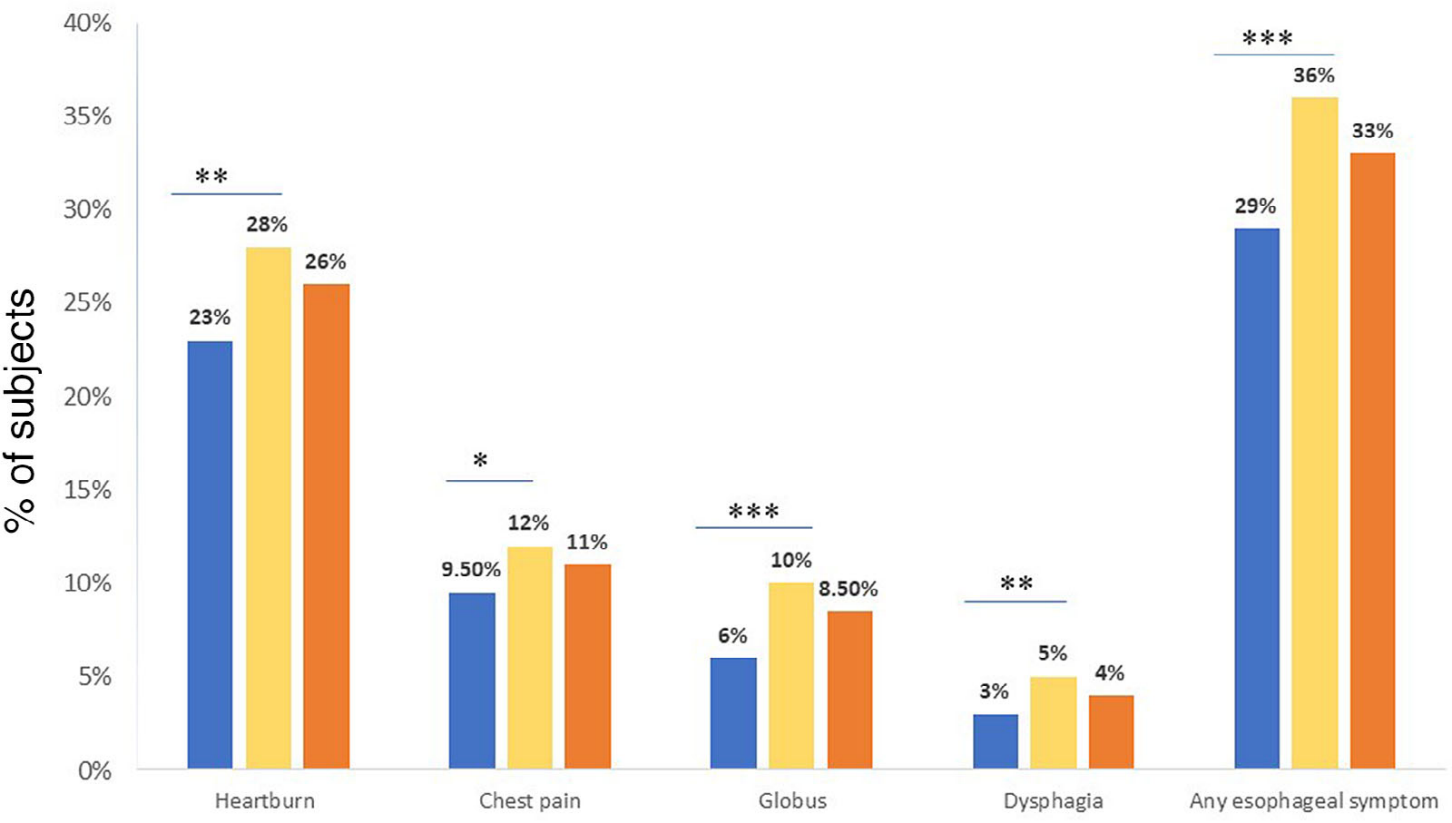
Gender‐specific and the overall prevalence of esophageal symptoms. (*P*‐value = *<0.05, **<0.01, ***<0.001). (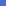), Male; (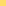), female; (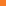), overall.

**Figure 3:**
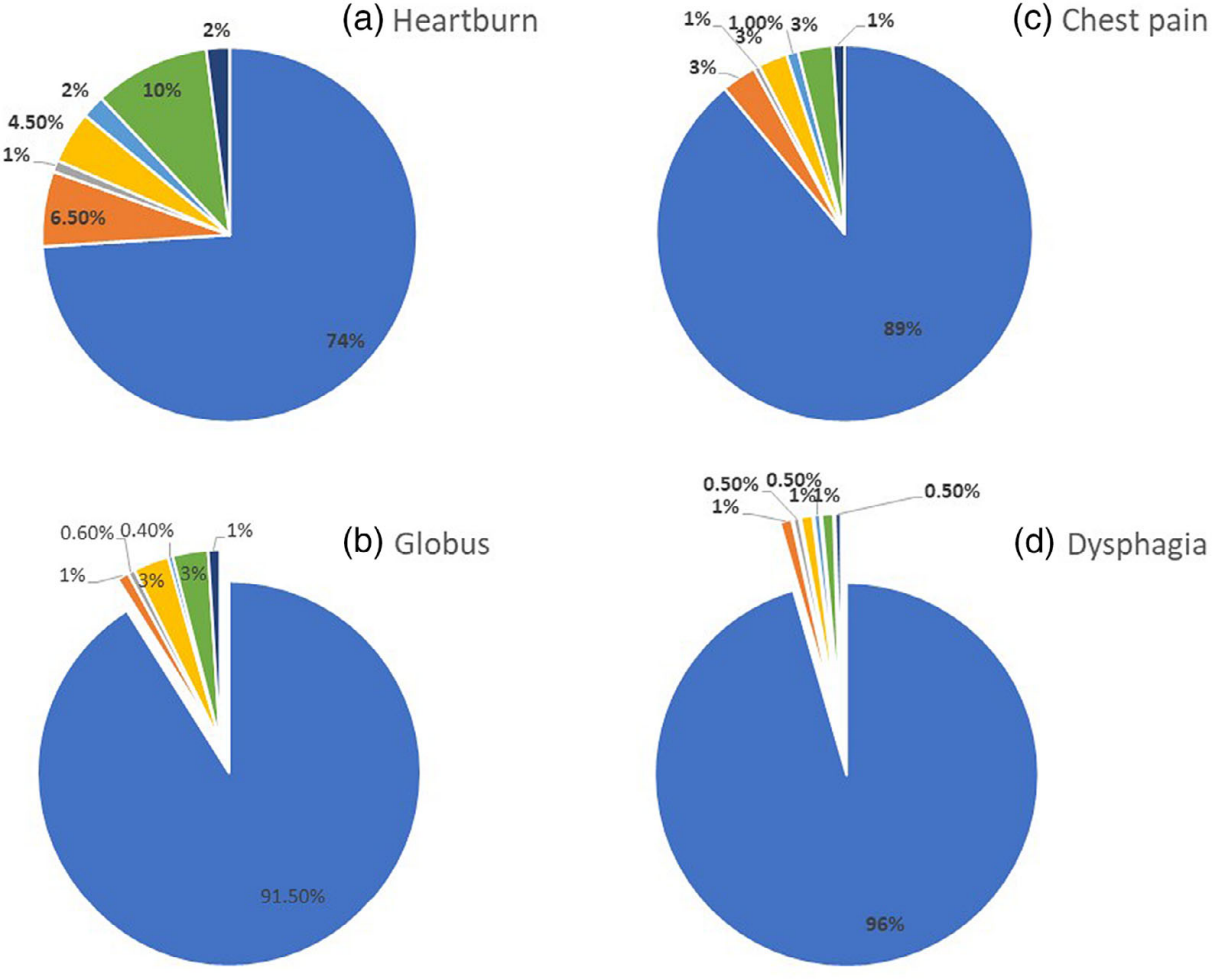
Frequency of esophageal symptoms. (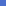), No symptoms; (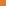), less than one day a month; (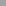), one day a month; (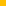), two or three days a month; (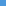), one day a week; (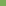), more than one day a week; (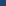), everyday.

Heartburn was the most frequent symptom, 863 (25.8%), followed by chest pain, 367 (11%); globus 285 (8.5%); and dysphagia, 146 (4.4%). Of the respondents, 1108 (33.1%) had at least one esophageal symptom. Of them, one, two, three, and all four symptoms were present in 711 (21.2%), 275 (8.2%), 88 (2.6%), and 34 (1%), respectively. Esophageal symptoms were more common among females compared to males, as shown in Figure 2. Symptom frequency is shown in Figure 3. Heartburn was present at a frequency of at least once a week in 465 (13.9%) subjects. By the frequency threshold and duration standards of Rome III criteria, 428 (12.8%), 41 (1.2%), 49 (1.5%), and 26 (0.8%) had heartburn, chest pain, globus, and dysphagia of presumed functional origin, respectively. At least one functional esophageal disorder was present in 524 (15.6%) subjects. The prevalence of esophageal disorders of presumed functional origin was 17.6% and 13% in females and males, respectively (<0.001).

### *Esophageal symptoms,* *FD* *only,* *IBS* *only, and* *FD‐IBS* *overlap*

Among the subjects with any esophageal symptoms, FD only was present in 25.9%, FD‐IBS overlap in 7.8%, and IBS only in 1.2% subjects (Table 1). Presence of FD only, FD‐IBS overlap, and IBS only was more common among subjects with heartburn, chest pain, dysphagia, and globus compared to subjects without any esophageal symptoms, as shown in Table S1. Overlap with FD only was most common among subjects with globus (48.1%), followed by chest pain (32.4%), dysphagia (29.5%), and heartburn (22.7%). Overlap with both FD and IBS was most common among subjects with dysphagia (19.2%), followed by globus (16.1%), chest pain (16.7%), and heartburn (8.6%). Subjects with heartburn had significantly higher FD only or FD‐IBS overlap but not IBS (Table S1).

**Table 1:** Sociodemographic, clinical, and psychological parameters of the subjects with and without esophageal symptoms

Characteristics	Subjects without esophageal symptoms (*n* = 2243)	Subjects with esophageal symptoms (*n* = 1108)	*P* value
Age, (years, mean ± sd)	39.82 ± 16.39	41.59 ± 15.25	0.002
Gender, *n* (%)			
Male	1012 (45.1)	415 (37.5)	<0.001
Female	1231 (54.9)	693 (62.5)	
Marital status, *n* (%)			0.123
Married	1775 (79.9)	903 (82.2)	
Single	446 (20.1)	196 (17.8)	
Education, *n* (%)			<0.001
Illiterate and up to class V	1141 (50.9)	657 (59.3)	
Class V and above	1102 (49.1)	451 (40.7)	
Family income†			
Lower family income	1388 (63.8)	808 (75)	<0.001
Higher family income	786 (36.2)	270 (25)	
Occupation, *n* (%)			
Housewife	1189 (61.2)	650 (63.7)	0.176
Cultivation	218 (11.2)	121 (11.9)	
Others	536 (27.6)	249 (24.9)	
Religion, *n* (%)			
Muslim	1479 (66.0)	742 (67.1)	0.519
Hindu	763 (34.0)	364 (32.9)	
Smoker (current or past), *n* (%)	363 (19.3)	188 (18.4)	0.546
Nonsmoker, *n* (%)	1520 (80.7)	836 (81.6)	
Presence of psychosocial stress, *n* (%) (Cut‐off value 23)	17 (0.8)	44 (4)	<0.001
Presence of FD‐IBS overlap, *n* (%)	24 (1.1)	86 (7.8)	<0.001
Presence of FD only, *n* (%)	150 (6.7)	287 (25.9)	<0.001
Presence of IBS only, *n* (%)	18 (0.8)	13 (1.2)	0.292
Somatic symptoms, (median score, interquartile range)	1.0 (0–2)	2.0 (1–3)	<0.001
Anxiety & insomnia, (median score, interquartile range)	0.0 (0–2)	0.0 (0–2)	0.001
Social dysfunction, (median score, interquartile range)	7.0 (4–7)	7.0 (5–7)	<0.001
Depression (median score, interquartile range)	0.0 (0–0)	0.(0–0)	<0.001
Total score (median score, interquartile range)	8 (6–9)	8 (7–11)	<0.001

† Lower family income≤ taka 10 000/month, higher family income ≥ taka 10 000/month.

FD, functional dyspepsia; IBS, irritable bowel syndrome.

### *Factors associated with esophageal symptoms*

Increasing age, female gender, education less than class V, lower family income, presence of psychological distress, and presence of FD‐IBS overlap and dyspepsia only were found to be associated with esophageal symptoms in the univariate analysis as shown in Table 1. The presence of IBS was not found to be associated with esophageal symptoms. The somatic symptom, social dysfunction, anxiety and insomnia, and the depression scores were higher among subjects with esophageal symptoms (all *P* < 0.05). The total GHQ‐28 score was also higher among these subjects, as shown in Table 1. On logistic regression analysis, female gender, lower family income, presence of psychological distress, presence of FD‐IBS overlap, and FD only were found to be risk factors for esophageal symptoms, as shown in Table 2.

**Table 2:** Logistic regression analysis for risk factors of esophageal symptoms

Characteristics	Adjusted odds ratio (OR)	95% confidence interval (CI)	*P*‐value
Increasing age	1.003	0.998–1.008	0.299
Gender			
Male	Reference		
Female	1.29	1.09–1.51	0.002
Marital status			
Single	Reference		
Married	0.985	0.803–1.21	0.889
Education			
More than class V	Reference		
Illiterate and up to class V	1.19	0.996–1.42	0.055
Family income†			
Higher family income	Reference		
Lower family income	1.45	1.20–1.74	<0.001
Presence of FD‐IBS overlap	5.36	2.63–10.93	<0.001
Presence of FD only	5.15	4.13–6.41	<0.001
Presence of IBS only	1.82	0.888–3.88	0.119
Presence of psychosocial stress (Cut‐off value 23)	2.56	1.32–4.9.	0.005

† Lower family income ≤ taka 10 000/month, higher family income ≥ taka 10 000/month.

FD, functional dyspepsia; IBS, irritable bowel syndrome.

### *Factors associated with heartburn*

On univariate analysis, increasing age, female gender, lower education status, lower family income, presence of FD‐IBS overlap, FD only, and psychological distress were associated with heartburn. On logistic regression analysis, lower family income, FD‐IBS overlap, FD only, and psychological distress were found to be risk factors for heartburn, as shown in Table 3.

**Table 3:** Logistic regression analysis for risk factors of heartburn

Characteristics	Unadjusted odds ratio, 95% confidence interval	*P* value	Adjusted odds ratio, 95% confidence interval	*P* value
Increasing age	1.007 (1.002–1.011)	0.003	1.005 (0.99–1.11)	0.76
Gender				
Male	Reference		Reference	
Female	1.37 (1.19–1.89)	<0.001	1.31 (0.94–1.83)	0.103
Marital status				
Single	Reference		Reference	
Married	1.15 (0.96–1.39)	0.12	0.85 (0.66–1.07)	0.17
Education				
More than class V	Reference		Reference	
Illiterate and up to class V	1.40 (1.21–1.63)	<0.001	1.17 (0.96–1.42)	0.11
Family income†				
Higher family income	Reference		Reference	
Lower family income	1.70 (1.44–1.99)	<0.001	1.46 (1.18–1.78)	<0.001
Occupation				
Housewife	Reference		Reference	
Cultivation	1.01 (0.079–1.29)	0.90	0.73 (0.51–1.03)	0.79
Others	0.85 (0.71–1.01)	0.07	0.91 (0.65–1.20)	0.58
Religion				
Hindu	Reference	0.51	Reference	
Muslim	1.05 (0.90–1.2)		0.87 (0.72–1.05)	0.15
Smoking				
Nonsmoker	Reference		Reference	
Smoker (current or past)	0.94 (0.78–1.1)	0.54	0.96 (0.73–1.24)	0.76
Presence of FD‐IBS overlap	7.78 (4.91–12.03)	<0.001	4.95 (3.90–6.28)	<0.001
Presence FD only	4.88 (3.44–6.03)	<0.001	4.95 (3.90–6.28)	<0.001
Presence of IBS only	1.46 (0.71–3.00)	0.294	1.77 (0.75–4.14)	0.18
Presence of psychosocial stress‡	5.42 (3.08–9.52)	<0.001	2.87 (1.43–5.75)	0.003

† Lower family income ≤ taka 10 000/month, higher family income ≥ taka 10 000/month.

‡ General Health Questionnaire 28‐ score 23 or more.

FD, functional dyspepsia; IBS, irritable bowel syndrome.

### *Esophageal symptoms, healthcare utilization, and quality of life*

#### *Consultation*

**Figure 4:**
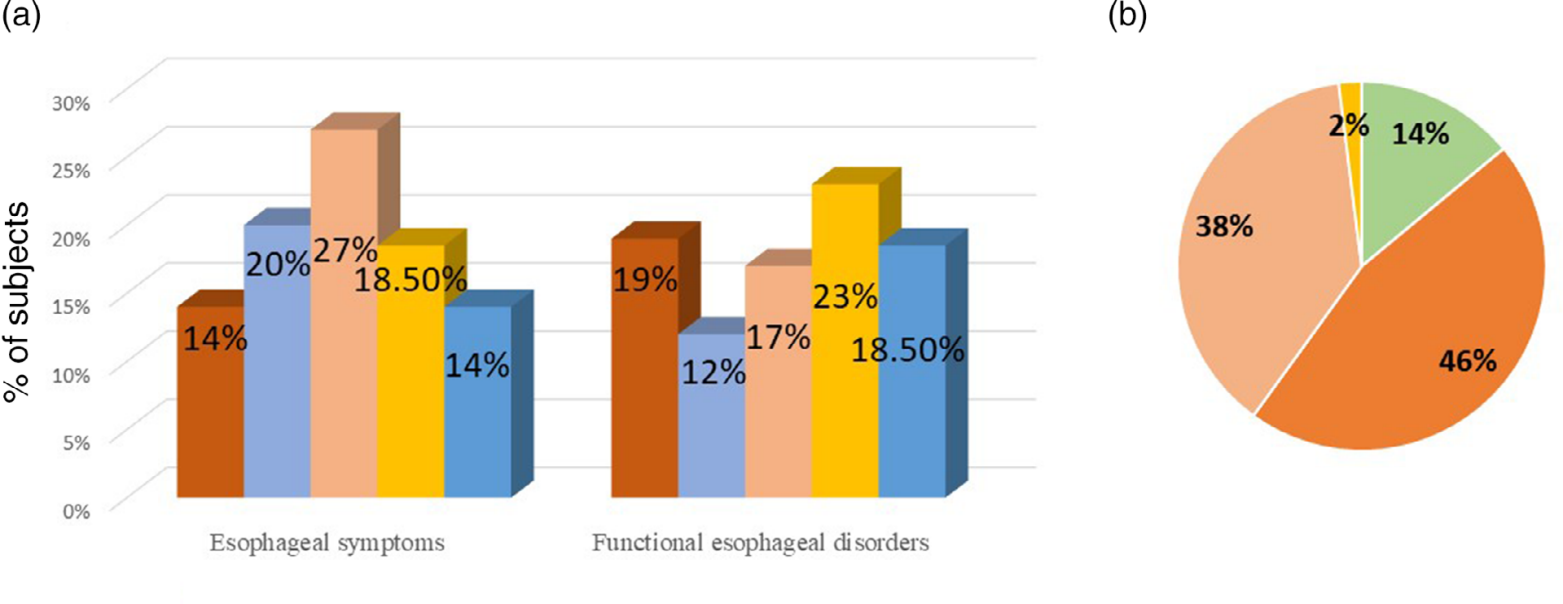
(a) Consultations for esophageal symptoms. (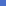), Heartburn; (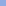), globus; (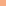), chest pain; (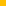), dysphagia; (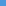), overall. (b) Satisfaction with treatments. (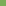), Satisfied; (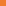), not satisfied due to lack of explanation; (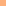), not satisfied due to lack of investigations; (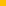), not satisfied due to lack of medications.

Figure 4a shows the consultation rates for different symptoms of presumed esophageal origin and the consultation rates of different esophageal disorders of presumed functional origin. Among 1108 individuals with any esophageal disorders and 2243 subjects without esophageal disorders, 156 (14.1%) and 71 (3.2%) consulted any health‐care provider for their problems, respectively (*P* < 0.001). Of 534 subjects considered to have presumed functional esophageal disorders based‐on Rome III criteria, 97 (18.5%) consulted healthcare provider for their problems.

#### *Medication use*

Among the subjects with and without esophageal symptoms, 517 of 1108 (46.6%) and 237 of 2243 (10.5%) subjects took a proton pump inhibitor (PPI) or Histamine 2 receptor antagonist (H2RA) in the last 3 months for their problems (<*P* = 0.001). Among subjects with esophageal symptoms, 429 (38.8%) took PPI, and 88 (7.9%) took H2RA. Data were obtained from 774 subjects with esophageal symptoms regarding the satisfaction of treatment. Only 107 (13.8%) were satisfied, and 667 (84%) were dissatisfied. Regarding the reasons for dissatisfaction, 355 (45.9%) subjects reported that they did not get enough explanation, 292 (37.7%) reported that they did not get enough investigations, and only 20 (2.6%) thought that they did not get enough medications, as shown in Figure 4b.

#### *Quality of life*

Of 863 subjects with heartburn, QoL was not affected at all in 295 (34%), a little in 210 (24%), somewhat in 125 (15%), a lot in 208 (24%), and a great deal in 25 (3%) subjects. Considering a lot and a great deal as the significant impairment of QoL, 233 (27%) of the subjects had impaired QoL due to heartburn.

## Discussion

This cross‐sectional study conducted among the adult population in a rural community of Bangladesh found that about 26% of the population had heartburn, 11% chest pain, 8% globus, and 4% had dysphagia. One‐third of the study population had at least one esophageal symptom. About one in six persons consulted health‐care providers, and half of the subjects took antisecretory drugs in the last 3 months for their esophageal symptoms. About a quarter of the subjects with heartburn had significant impairment of QoL.

The findings of the study suggest a considerable burden of esophageal symptoms in the rural community of Bangladesh. A hospital‐based multinational study in Asia found that 28.1% of 1532 FGID subjects had at least one esophageal symptom, although the prevalence varied from 17.6% in China to 37.5% in India.^1^ A recent internet survey conducted in the United States, Canada, and United Kingdom using the Rome IV questionnaire found that the prevalence of globus, heartburn, dysphagia, and chest pain was 8.1, 6.5, 5.2, and 4.5%, respectively. At least one esophageal symptom was present in 17% of subjects.^11^ In a recently published global study on FGIDs in 33 countries, using the Rome IV questionnaire, the prevalence of globus, heartburn, dysphagia, chest pain, reflux hypersensitivity, and at least one esophageal disorder was 0.8, 1.1, 3.2, 1.4, 0.8, and 6% and 0.2, 0.4, 1.2, 1, 0.6, and 2.9% by internet survey and household survey, respectively.^19^ Although the findings of the prevalence of globus and dysphagia are comparable to the present study, heartburn frequency and at least one esophageal symptom are different. There may be at least three reasons for such differences. First, the surveys excluded the subjects who admitted that they were previously diagnosed with GERD for their symptoms. Although the studies did not mention the basis of diagnosis and exclusion of GERD, the inclusion of such subjects in the analysis would increase the prevalence of heartburn and esophageal symptoms. Second, the diagnostic criteria changes, mainly the frequency threshold for diagnosis of esophageal symptoms between Rome III and Rome IV criteria, might influence the results. The frequency threshold for diagnosis by Rome III *versus* Rome IV for functional heartburn is at least once a week *versus* at least twice a week; for functional chest pain of presumed esophageal origin, 2–3 times/month *versus* at least once a week; for globus, more than once a month *versus* at least once a week; and for functional dysphagia, at least once a month *versus* at least once a week. The third reason could be related to the fact that only one subject per family was included in the Rome Foundation global epidemiology survey. As most FGIDs show familial clustering, such a design might have underestimated the burden. There are a few studies on the epidemiology of functional heartburn.

Among the patients with reflux symptoms such as heartburn, 70–80% have no endoscopic evidence of erosive esophagitis. Such patients have either nonerosive reflux disease (NERD), reflux hypersensitivity, or functional heartburn.^20^, ^21^ Clinic‐based studies suggest that the prevalence of functional heartburn varies from 35 to 75% among patients with reflux symptoms.^22^, ^23^, ^24^ A recent review showed that as much as 21–39% of patients with heartburn refractory to PPI undergoing pH impedance monitoring meet the criteria for functional heartburn.^25^ However, the community‐based data on what proportion of patients with heartburn have erosive esophagitis, NERD, reflux hypersensitivity, or functional heartburn are lacking.

In our study, 11% of the population had chest pain of presumed esophageal origin. In a household survey in the United States, 13.6% of the subjects had functional chest pain.^26^ As this is a diagnosis of exclusion, the exact prevalence of NCCP is unknown. A meta‐analysis of community‐based studies on NCCP found a pooled prevalence of 13% with lower prevalence with Rome I and II criteria.^27^ NCCP includes GERD, eosinophilic esophagitis, and esophageal motor disorders in addition to functional chest pain. It is estimated that, among the patients with NCCP, 50–60% had GERD, 15–18% had esophageal dysmotility, and only 32–35% had actual functional chest pain.^28^

Globus is common in clinical practice,^29^ but there are limited data on its prevalence in the community. In our study, globus was present in 8% of the population. In a population‐based study using a structured questionnaire, 16% of 337 subjects had globus.^10^ This difference might result from differences in the definition. Globus may result from the structural lesion or motor abnormalities or may be idiopathic. In clinical practice, the simultaneous presence of throat pain, weight loss, or odynophagia warrants investigations such as upper GI endoscopy.^6^ The frequency of idiopathic globus in the community has not been studied after excluding structural disease or motor disorders by investigations.

Dysphagia was the least prevalent esophageal symptom (4%) in the present study, which is similar to the findings of the household survey in the United States, where 4% of the population had dysphagia.^26^ There is no community‐based data on the frequency of functional dysphagia. In a tertiary center in the United State, regarding the diagnostic yield in the evaluation of dysphagia among 694 patients, functional dysphagia was present in 2.3% and 11.2% of the subjects with normal endoscopy.^30^

The somatic symptoms, social dysfunction, anxiety and insomnia, and the depression scores were higher among the subjects with esophageal symptoms in the present study. Psychological distress has been found in association with different esophageal symptoms.^6^ Psychological stress can trigger esophageal symptoms.^31^ A high prevalence of psychological disorders, such as panic disorder, anxiety, and depression, has been found in patients with NCCP.^8^ Co‐existing psychological factors have been reported in patients with functional heartburn.^32^ Life stress is associated with symptom onset and exacerbations in globus.^33^

In this study, the presence of FD‐IBS overlap and FD only were independent predictors of esophageal symptoms. The population‐based online survey from the United States, United Kingdom, and Canada found that an increased number of regions for FGIDs (gastroduodenal, bowel, and anorectal) increased the proportion of subjects with esophageal symptoms significantly.^11^ Functional heartburn has been reported to be associated with functional dyspepsia^34^ and IBS.^35^, ^36^, ^37^ The hospital‐based study in Asia found an association between esophageal symptoms and IBS‐C and FD.^1^ But these studies did not compare the association between functional heartburn and FD alone, FD‐IBS overlap, and IBS alone. The present study suggests that the overlap of heartburn with IBS may result from the overlap of IBS with FD rather than IBS alone. The overlap of esophageal symptoms with FD or IBS may result from shared common pathophysiological mechanisms such as excessive acid exposure, visceral hypersensitivity, and psychological abnormalities.

This study conducted among a sizeable rural population using a translated and validated questionnaire demonstrated a considerable burden of esophageal symptoms, which adversely affects the QoL and is associated with considerable health resources utilization. These findings had significant implications in allocating health‐care resources and essential variables in determining the outcome measures in clinical trials. The finding that about one‐third of the patients of esophageal symptoms had overlap with FD or IBS or both suggest the importance of routine inquiries about symptoms of FD and IBS in a patient with esophageal symptoms in clinical practice.

Bangladesh is a small country where most of the people live in the villages. It is homogenous in terms of dietary habits and sociocultural perspectives. Thus, this study may well represent the burden of esophageal symptoms in the country. One of the limitations of this study is the usage of Rome III rather than currently iterated Rome IV criteria. Rome III criteria was used because the study was conducted before the publication of the Rome IV criteria. Another limitation is that investigations such as endoscopy of UGIT, esophageal manometry, and pH monitoring and inquiries on the presence of organic disease had not been carried out to exclude the structural, mucosal, and motor disorders to estimate the burden of functional esophageal disorders. We estimated the prevalence of esophageal symptoms of a 3‐month duration with a different frequency of occurrences due to the inability to conduct such investigations in the community. Besides, we estimated the frequency of esophageal disorders by Rome III criteria with its requirements of at least 3 months of symptoms, frequency threshold, and onset occurring 6 months before diagnosis.

In conclusion, esophageal symptoms are common in the rural community of Bangladesh, have a negative impact on QoL, and are associated with substantial health resource utilization. Lower family income, the presence of psychological distress, FD‐IBS overlap, and the presence of FD were the risk factors of esophageal symptoms. Further studies are needed to determine the true prevalence of structural lesions, motor disorders, and functional esophageal disorders in the community.

## Supporting information


**Table S1.** Overlap of esophageal symptoms with functional dyspepsia (FD), irritable bowel syndrome (IBS), and FD‐IBS overlap.
