# Supplementary material for: Prevalence, risk factors, and healthcare‐seeking among subjects with esophageal symptoms: A community‐based study in a rural Bangladeshi population
Source: JGH Open. 2020 Sep 22;4(6):1167–75. doi: 10.1002/jgh3.12417 (PMC7731836; doi:10.1002/jgh3.12417)
Supplement: Supplementary file 1 — Table S1. Overlap of esophageal symptoms with functional dyspepsia (FD), irritable bowel syndrome (IBS), and FD‐IBS overlap. [file JGH3-4-1167-s001.docx]

|  | **Heartburn** | | | **Chest pain** | | | **Dysphagia** | | | **Globus** | | |
| --- | --- | --- | --- | --- | --- | --- | --- | --- | --- | --- | --- | --- |
| **Overlap** | **Present**  **(Total =863)**  **n (%)** | **Absent**  **(n=2488)**  **n (%)** | **P value** | **Present**  **(n=367)**  **n (%)** | **Absent**  **(n=2488)**  **n (%)** | ***P***  **value** | **Present**  **(n=146)**  **n (%)** | **Absent**  **(n=3205)**  **n (%)** | ***P* value** | **Present**  **(n=285)**  **n (%)** | **Absent**  **(n=3066)**  **n (%)** | ***P* value** |
| FD only | 196(22.7) | 241 (9.7) | <0.001 | 119(32.4) | 318(10.7) | <0.001 | 43(29.5%) | 394(12.3) | <0.001 | 137(48.1) | 300(9.8) | <0.001 |
| FD-IBS overlap | 74 (8.6) | 36(1.4) | <0.001 | 62(16.7) | 48(1.6) | <0.001 | 28(19.2) | 82(2.6) | <0.001 | 46(16.1) | 64(2.1) | <0.001 |
| IBS only | 12(1.4) | 19(0.8) | 0.102 | 8(2.2) | 23(0.8) | 0.016 | 4(2.7) | 27(0.8) | 0.04 | 6(2.1) | 25(0.8) | 0.043 |

Supplementary Table 1: Overlap of esophageal symptoms with functional dyspepsia (FD), irritable bowel syndrome (IBS) and FD-IBS overlap
